# 10 years of research: from ignoring Modic changes to considerations regarding treatment and prevention of low-grade disc infections

**DOI:** 10.4155/fsoa-2016-0017

**Published:** 2016-04-05

**Authors:** Claus Manniche, Alan Jordan

**Affiliations:** 1 Spine Center of Southern Denmark & University of Southern Denmark, Oestre Hougvej 55, 5500 Middelfart, Denmark; 2 Broadgate Spine & Joint Clinic, 65 London Wall, London, EC2M 5TU, UK

**Keywords:** disc infection, low back pain, Modic changes

**Figure 1.**
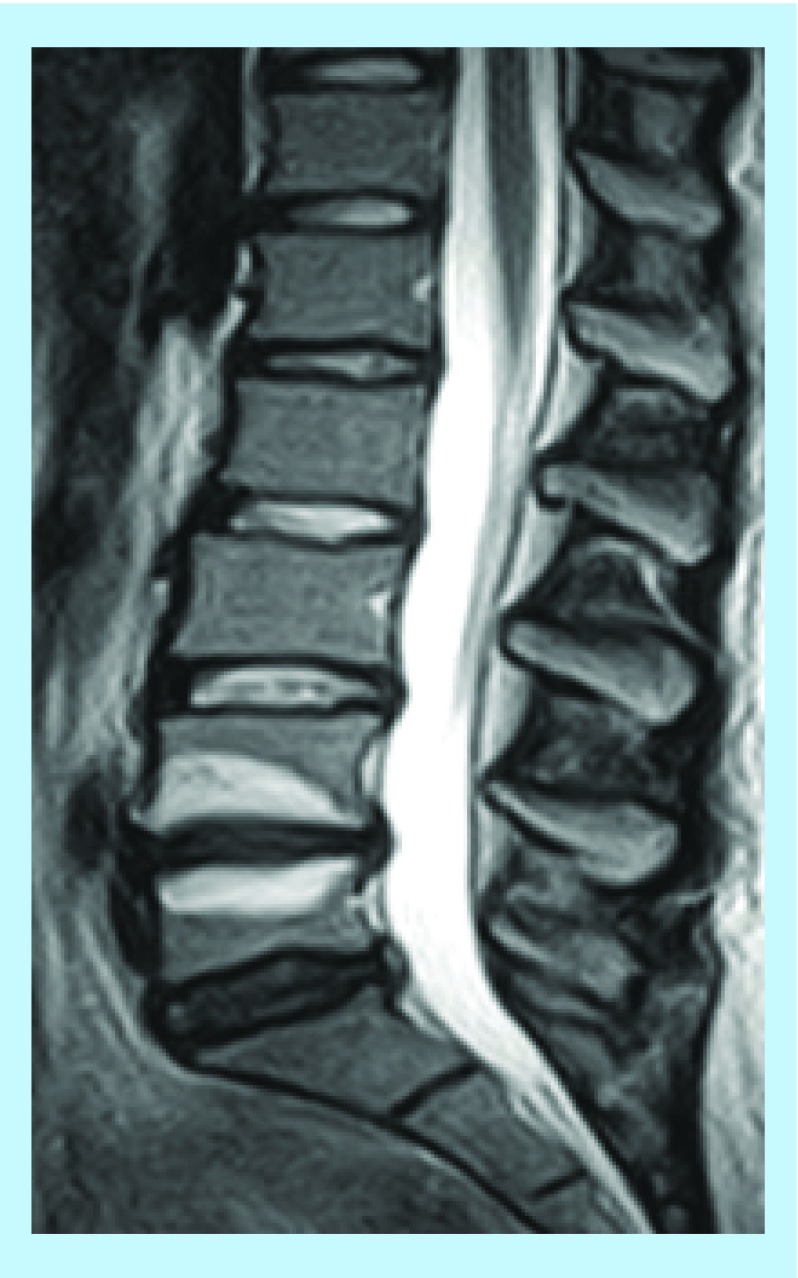
**An MRI T2-weighted image.** Typical Modic changes type 1 (high signal intensity) in a lumbar segment. Reproduced with permission from [4].

**Figure 2.**
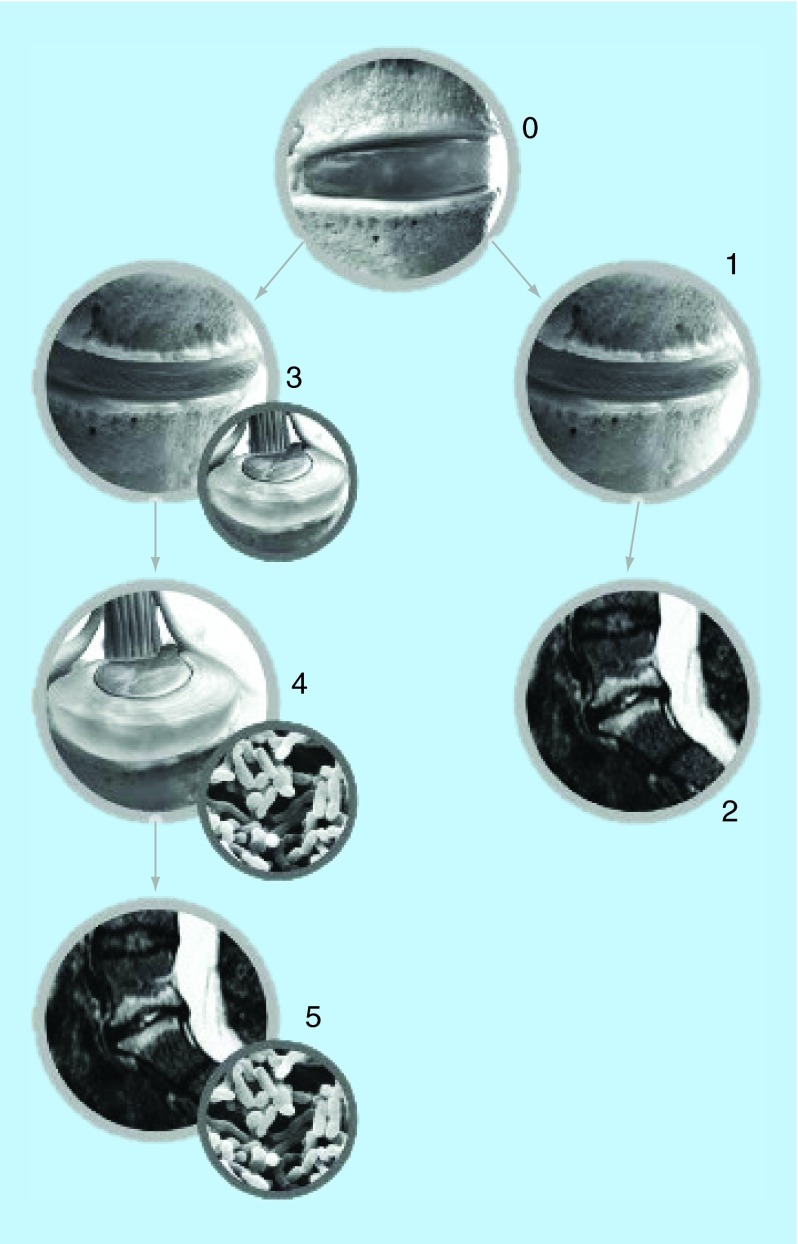
**The development of Modic changes.** Two associated pathways. Mechanical pathway: **(0)** normal disc; **(1)** age-related disc degeneration and the development of spondylosis; **(2)** end plate damage with development of Modic changes type 1. Infectious pathway: **(3)** disc degeneration with the development of a disc herniation; **(4)** invasion of bacteria, causing a disc infection; **(5)** end plate damage and bacteria with the development of Modic changes type 1. Reproduced with permission from [8].

After many years of essentially ignoring or considering Modic changes (MC) as seen on MRI scans as incidental findings, a new era began with the publication of Kjær *et al*. 10 years ago, who were the first research group in the world to demonstrate a clear correlation between MC and the 1-year prevalence of back pain [1]. This important discovery ‘upset’ the prevailing consensus among back specialists that back pain was predominantly a biopsychosocial phenomenon with an emphasis on the psychosocial dimension [2]. Since Kjær *et al.'s* publication, the biological dimension of back pain has become a hot topic among back pain researchers throughout the world. Currently, new publications dealing with Modic-related topics are published almost weekly in scientific journals.

We will introduce the reader to the concept of MC and present the current status of our knowledge from the very latest scientific literature including the presence of *Propionibacterium acnes* inside the disc. Many of the bricks involved in this puzzle are beginning to form a biologically sound ‘picture’.

## Definitions, pathoanatomy/genesis, symptoms & treatment

In the literature [3,4], three types of MC are defined; MC type 1, type 2 and type 3. Type 1 is characterized by high signal intensity on T2-weighted images (Figure 1) and low signal intensity on T1-weighted images. The extent of each MC can vary from a narrow fringe that simply involves the end plate of the vertebra to spreading into large parts of the adjacent vertebra. MC type 2 is characterized by high signal intensity on both T1- and T2-weighted images. MC type 1 contains vascular tissue and active inflammatory agents; type 2 contains granulation tissue infiltrated by significant amounts of fat cells. This has been demonstrated in biopsy studies. MC Type 3 are rare and do not appear to have any clinical relevance. They represent subchondral bony sclerosis with low T1 and T2 signal intensity. In patients with prolonged back pain, the prevalence of MC is 40%. In individuals with MC, more than 90% will have back pain within 1 year. MC often causes localized pain 24/7 and nocturnal pain is the rule rather than the exception [5]. A direct correlation between the size of the MCs as seen over time and pain intensity variations has been demonstrated [6]. MC type 1 are an important prognostic marker of a poor prognosis regarding relentless pain and reduced daily function [6]. We refer to references [4] and [7] for additional detail.

The most common cause of MC may well be that the ordinary disc degenerative processes involving damage and microfractures to the vertebral end plates (Figure 2; nr. 0-1-2). In some patients, the degenerative process results in the development of edema and inflammation of the adjacent vertebrae. Several factors may be involved: end plate damage following disc herniation, ingrowth of capillaries and inflammatory agents related to the end plate structure may be important ingredients in the development of pain and MC in the involved spinal segment [9]. As the bony end plates normally have a rich supply of small free nerve endings, degenerative developments and inflammation likely trigger localized inflammatory pain [9].

Following disc herniations or annular tears, bacteria may enter the disc and initiate a discitis (Figure 2: nr. 0-3-4-5) [4]. Low or high virulent infections may take place depending upon the bacterial species or subtype [10]. Anaerobic *P. acnes* bacteria are often present in the herniated mass following an acute disc herniation [4].

The degenerative and the infectious pathways may work concurrently and together accelerate the destruction of the spinal segment structures [9,11].

In most cases, treatment is similar to that recommended for other cases of disc degeneration/spondylosis that are not spontaneously improving. In most cases, no relief is seen following back exercises or general training [4]. If there is a suspicion of a disc infection, prolonged antibiotic therapy may be an effective treatment. In 2013, a randomized clinical trial (RCT) with 1-year follow-up RCT investigated the efficacy of amoxicillin/clavulanic acid for a 3-month period in patients with disc herniations and MC type 1 as seen on MRI [12]. In more than 50–60% of these patients clinically relevant and statistically significant improvements were seen at the end of treatment with further improvement in the actively treated group up to 1-year follow-up. Another smaller RCT with no follow-up period also demonstrated a positive clinical effect of antibiotic treatment in patients with MC type 1 [13]. Several large RCTs are underway or in the planning stages in Norway, Australia and the UK.

## The infectious pathway: the scientific case

It has been acknowledged for many years that disc degeneration – typically initiated by trauma or local mechanical forces – involved the vertebral end plates during the progressive destructive phase, in some patients. The resulting end plate damage is associated with the MRI finding, MC [9–11].

In 2008, a Danish research group proposed the theory that in addition to the degenerative/mechanical pathway toward developing MC, an alternative infectious pathway likely took place concurrently [4]. The infectious pathway takes place as a result of a series of occurrences in and around the disc and end plates as is the case for the degenerative/mechanical pathway. The latest research in this field appears to confirm the existence of an infectious pathway:

Low virulent bacteria enter the disc following a herniation or small trauma related annular tears [11,14]. This process may well be facilitated by the tearing of end plate fragments often seen in biopsies of herniated material that render the end plate damaged, which also delays herniated disc material resorption and results in neovascularization [15–18];
The bacteria are randomly transported through the circulatory system to the discs or may enter from contamination in spinal-related surgical procedures [4,11,18];
When the skin barrier is broken, there is a risk of bacterial spreading from the folds of the skin and sebaceous glands to spine despite sterilization procedures [19]. In some cases, the invading bacteria may have a nonskin origination, for example, from the oral cavity, as shown by Rollason *et al*. in a phylogenetic study [20];
The most commonly found bacteria in the disc and herniated discal tissue are different subtypes of *P. acnes* [20–22];
Bacteria with sufficiently virulent characteristics are able to colonize intradiscally [10];
This is followed by inflammation and accelerated degeneration that in turn leads to end plate damage and MC changes as seen on MRI scans [10,23,24];
Disc-related inflammation will lead to back pain and the injection of anti-inflammatory agents has demonstrated short-term pain reduction in several studies [23,25];
A long-term antibiotic regimen results in a 50% reduction of pain in 50% of patients with low back pain following a disc herniation and Modic changes type 1 [12,13].

## Conclusion & future perspective

As the infectious pathway is shown to exist as an alternative to the degenerative/mechanical pathway, it is relevant to ask how we can differentiate the diagnoses ‘low virulent bacterial discitis/MC’ from ‘mechanical disc degeneration/MC?’ In which cases is antibiotic treatment relevant to consider? Which antibiotic regimes are most effective and provide the patients and the environment with the highest degree of protection? Are there other treatment alternatives? Can one imagine preventative steps which may prevent a bacterial infection through contamination?

There are currently no conclusive answers to these questions.

We must acknowledge that differing analytical methods used to identify which bacteria are found in discs are by no means effective. When the spread of findings ranges from 3 to 53% regarding the presence of bacteria in discal tissue [11], this is likely due to poor and difficult analytical methods. More readily available and reliable analytical methods are required. Perhaps it may be possible to develop blood tests that are sensitive for intradiscal bacterial infection?

The MRI-based diagnostics are not completed yet. End plate damage has not been standardized [4,26] and the interpretation of MC types 1 and 2 are often based upon the radiologists’ subjective evaluation.

It seems appropriate to treat patients with MC type 1. It is, therefore, imperative to continue researching the utilization of antibiotic treatment for this condition. Which treatment regimen is most effective? Is perioperative preventative antibiotic treatment relevant in the prevention of low virulent discitis?

*P. acnes* beyond the skin may result in many different serious opportunistic infections: in bone, implants, glands, lungs and vascular tissues, and there are still many unanswered questions before puzzle has been completed [27].

The current increased documentation of an infectious pathway in disc disease has increased our need to address the related questions not least due to the fact that appropriate treatment forms for this condition already seems to be visible in the horizon.
